# Influence of Different Drilling Protocols for Primary Stability at Low‐Density Bone Detected by Bone Density Measurement Drill

**DOI:** 10.1002/cre2.70430

**Published:** 2026-08-20

**Authors:** Yusuke Morimoto, Reiko Kobatake, Kazuya Doi, Yoshifumi Oki, Kaien Wakamatsu, Tomoko Izumikawa, Kazuhiro Tsuga

**Affiliations:** ^1^ Department of Advanced Prosthodontics Hiroshima University Graduate School of Biomedical and Health Sciences Hiroshima Japan

**Keywords:** bone density, cancellous bone, dental implants

## Abstract

**Objectives:**

Intraoperative bone assessment is critical for achieving primary implant stability, particularly at low‐density bone sites. Drilling torque value (DTV) has been proposed as an indicator of bone density and may help guide drilling preparation protocols to improve primary stability. However, the relationship between DTV and primary stability, especially histological bone parameters in biological bone, remains unclear. This study aimed to clarify the influence of different drilling protocols for primary stability at low‐density bone detected by DTVs in biological cancellous bones.

**Materials and Methods:**

Cancellous bone blocks obtained from bovine ribs were used. A specially designed torque measurement drill was used to assess bone density intraoperatively. Based on the DTVs, low‐density bone sites corresponding to Misch D4 and D5 were selected. The dental Implants were placed using either a normal or undersized drilling protocol. The DTVs, insertion torque values (ITVs), and implant stability quotient (ISQ) were recorded. Histological evaluation was performed to assess the bone area fraction (BAF) around the implant.

**Results:**

The ITVs were lower than the clinically recommended range for primary stability in both groups; however, they were significantly higher in the undersized group than in the normal‐sized group. The DTVs positively correlated with the ITVs in both groups. A positive correlation between DTV and ISQ was observed only in the normal‐sized group, whereas ITV was positively correlated with ISQ in both groups. Histological analysis revealed no significant difference in BAF between the two groups. BAF positively correlated with DTV, ITV, and ISQ.

**Conclusions:**

DTV reflects the existing bone volume of low‐density tissue and is correlated with primary stability. Intraoperative assessment of drilling torque would facilitate the selection of appropriate drilling protocols to achieve primary stability. Bone density evaluation by measuring the DTV may provide a clinically useful method for the intraoperative evaluation at low‐density implant sites.

**Abbreviations**
BAF: bone area fraction
CBCT: cone‐beam computer tomography
DTV: drilling torque value
ISQ: implant stability quotient
ITV: insertion torque value
ROI: region of interest

## 1 Introduction

The success of dental implant treatment is influenced by multiple factors, among which the bone density at the implant placement site is considered one of the most critical determinants of clinical outcomes (Marquezan et al. 2012). In sites with low bone density, sufficient mechanical contact between the implant and surrounding bone may not be achieved, resulting in inadequate primary stability (Turkyilmaz and McGlumphy 2008). Insufficient primary stability increases the risk of micromotion during the healing period, which may impair osseointegration and, in severe cases, lead to implant failure (Trisi et al. 2009). In cases where bone density is low and adequate primary stability cannot be achieved using a conventional drilling protocol, an undersized drilling technique—whereby the drilling protocol is intentionally prepared to be smaller than the implant diameter—may be employed to enhance primary stability through bone compression (Alghamdi et al. 2011; Orak et al. 2025). Conversely, in sites with high bone density, excessive heat generation during drilling and implant insertion may occur, potentially leading to thermal bone necrosis. Thus, the use of a tapping drill or modification of the drilling protocol may be required (Eriksson and Albrektsson 1983). Therefore, selecting an appropriate drilling protocol based on the bone density at the implant site is essential for safe and predictable implant treatment.

Various bone density assessments are performed in clinical practice. Cone‐beam computed tomography (CBCT) is widely used in dental practice; however, unlike multidetector CT, CBCT does not allow reliable quantitative evaluation of bone density using Hounsfield units (Gaur et al. 2022; Pauwels et al. 2015). Additionally, the intraoperative assessment of bone condition based on the surgeon's tactile sensation during drilling is subjective and highly dependent on clinical experience, leading to potential variability and limited reproducibility (Rokn et al. 2018; Linck et al. 2016). Consequently, a direct and objective evaluation of bone density to guide the selection of an optimal surgical protocol remains difficult in routine clinical practice.

To address this problem, we developed a novel bone density measurement drill and established a method for intraoperative bone density evaluation of bone cutting torque (Wakamatsu et al. 2024). This drill can be used without interfering with the implant placement protocol and enables objective assessment of bone density by measuring drilling torque values (DTVs) prior to implant socket preparation.

In previous studies using solid rigid polyurethane bone blocks corresponding to Misch bone classifications D2 to D4, a significant correlation was observed between the DTV and the insertion torque value (ITV) of dental implants (Misch 1990; Wakamatsu et al. 2024). Furthermore, the application of undersized drilling under low‐bone‐density conditions results in a significant increase in ITV, suggesting that DTV measurement may serve as a useful indicator for achieving adequate primary stability (Morimoto et al. 2025).

However, these artificial bone models have a uniform density structure that differs from biological bone, which has a three‐dimensional structure. Furthermore, bone structure is heterogeneous and consists of both cortical and cancellous bone, with substantial variations in thickness and density depending on the anatomical site (Osterhoff et al. 2016; Chirchir 2016). While sufficient primary stability can generally be obtained in sites with thick cortical bone, implant stability in sites with thin cortical bone relies predominantly on the cancellous bone, making bone density in the cancellous region particularly critical (Al‐Juboori et al. 2024; Miyamoto et al. 2005; Hsu et al. 2013).

Therefore, the present study aimed to clarify the influence of different drilling protocols on the primary stability of low‐density bone as detected by DTVs in biological cancellous bones.

## 2 Materials and Methods

### 2.1 Materials and Experimental Overview

**Figure 1:**
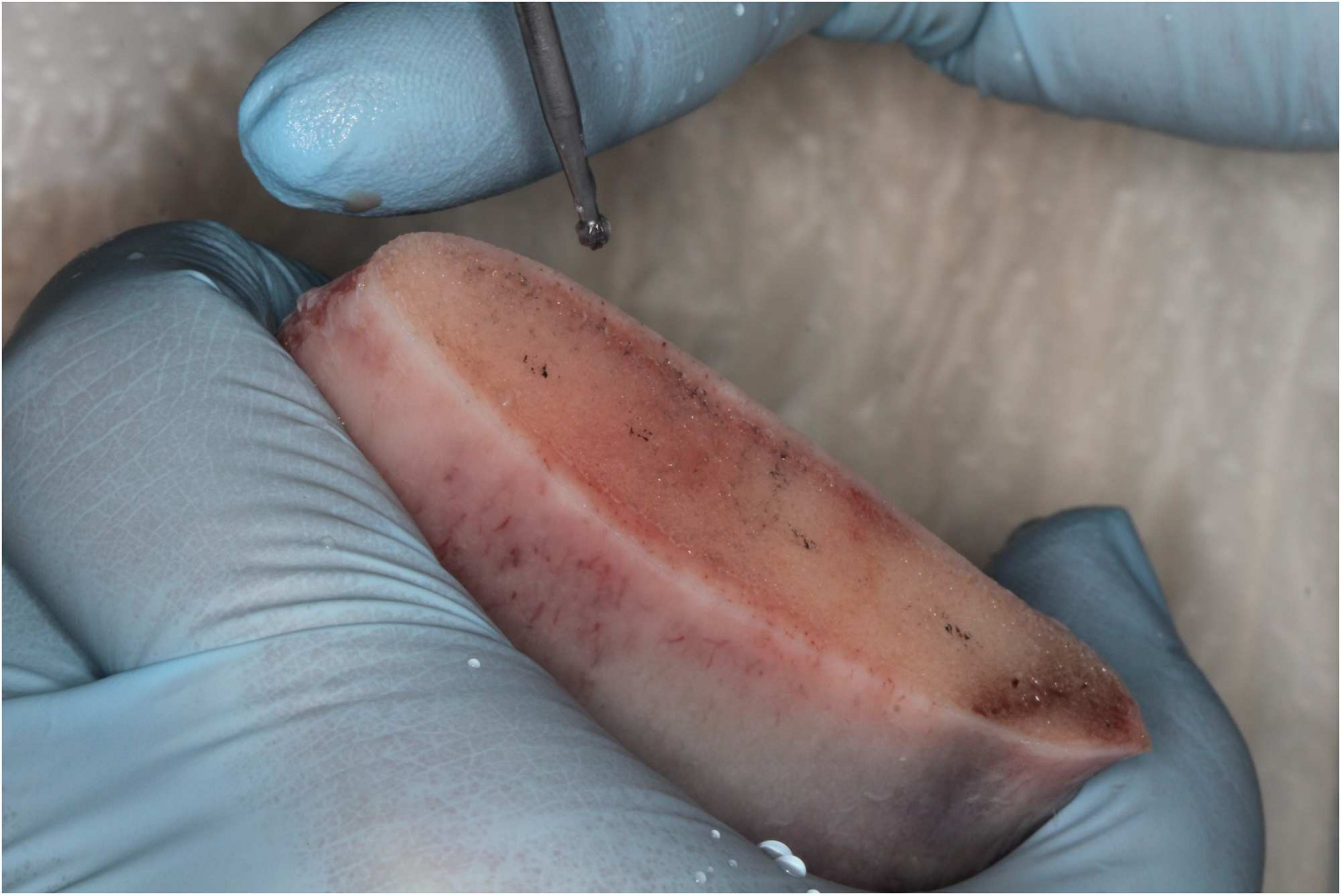
Bovine ribs.

Frozen, non‐fixed bovine rib sections, specifically cancellous bone portions, were used (Figure 1). Low‐density bone sites corresponding to Misch D4–D5 were selected based on bone density classification using drilling torque measurements, and the DTV was recorded (Doi et al. 2025). The measurement sites were set at 1 cm intervals. Implant sockets were prepared for undersized and normal‐sized drilling at the measurement sites, and tapered dental implants (4.2 mm diameter × 10 mm length, machined surface, FINESIA, Kyocera, Japan) were placed. The ITV was recorded as the primary stability. DTV measurements and implant socket preparation were performed using an oral surgery micromotor system (Surgic Pro2, Nakanishi, Japan). It has the advantage of enabling torque evaluation at 0.05‐s intervals with a resolution of 0.1 Ncm (Kobatake et al. 2025). The implant placement and implant stability quotient (ISQ) were measured using a resonance frequency analysis (Osstell ISQ, Osstell AB, Sweden). Measurements in both groups were performed at sites with a similar DTV on the same bone block　(*n* = 18 per group).

Markings were made at 1‐cm intervals on the cancellous bone area of the cut surface.

The upper bar represents the undersized group, and the lower bar represents the normal‐sized group. Following measurement of the DTV, implant socket preparation was performed in the undersized and normal‐sized groups, after which implant placement and primary stability evaluation were conducted.

### 2.2 Drilling Torque Measurement

The DTV was measured following previously reported protocols (Wakamatsu et al. 2024; Doi et al. 2025):

1. φ2.0 mm round‐shaped drill, 1200 rpm
2. φ2.0 mm twist drill, 1200 rpm, depth 10 mm
3. φ2.8 mm pilot drill, 1200 rpm, depth 4 mm
4. φ2.7 mm measurement drill, 35 rpm, measurement depth 3 mm

All procedures were performed under irrigation. The peak value obtained was recorded.

### 2.3 Implant Socket Preparation

**Figure 2:**
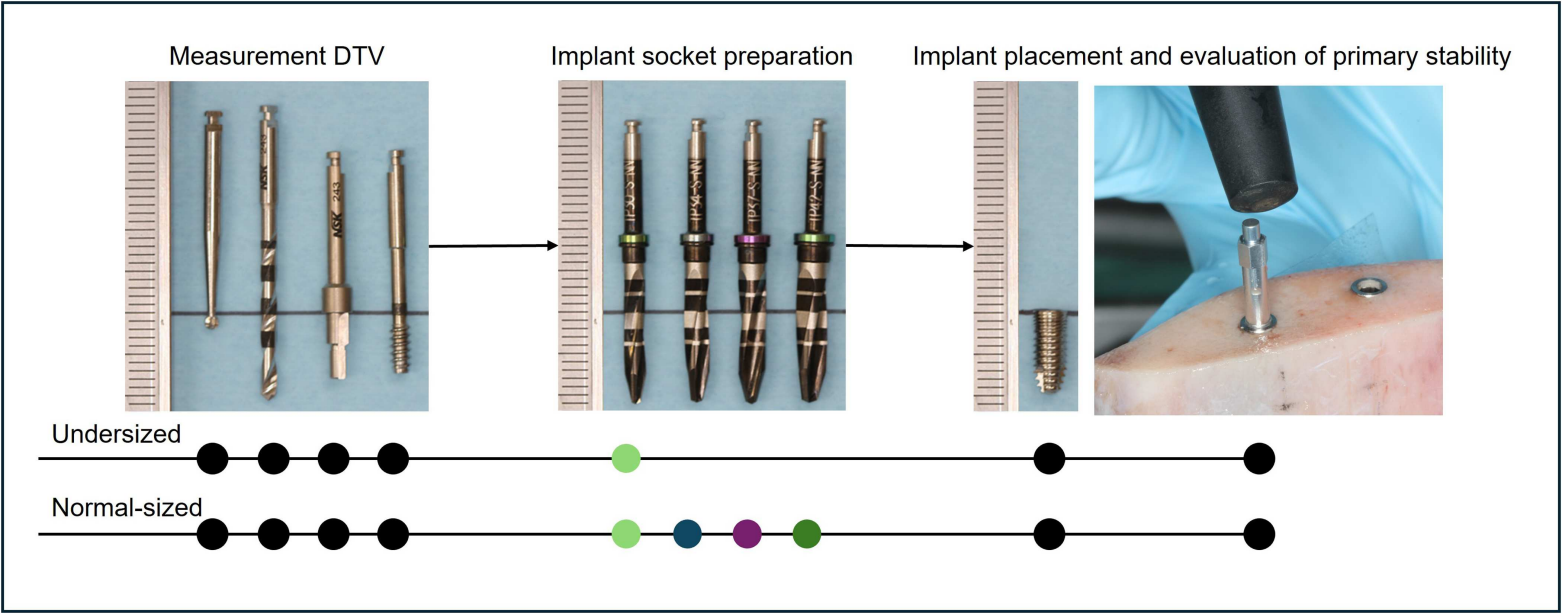
Experimental overview.

Implant socket preparation was performed for two groups (Figure 2):

Undersized group: Only a φ2.7 mm drill was used as the final drill under irrigation at 1200 rpm to a depth of 10 mm.

Normal‐sized group: Final drilling was performed sequentially using φ2.7 mm, φ3.1 mm, φ3.4 mm, and φ3.9 mm drills under irrigation at 1200 rpm to a depth of 10 mm.

### 2.4 Implant Placement and Primary Stability Evaluation

Implants were inserted at 35 rpm, and the peak insertion torque was recorded using a torque limiter set at 50 Ncm. Following insertion, a SmartPeg (Osstell AB, Sweden) was attached to each implant to measure the ISQ. The ISQ was measured three times for both the long and short axes, and the average of the two axes was recorded as the ISQ for each implant.

### 2.5 Histological Analysis

**Figure 3:**
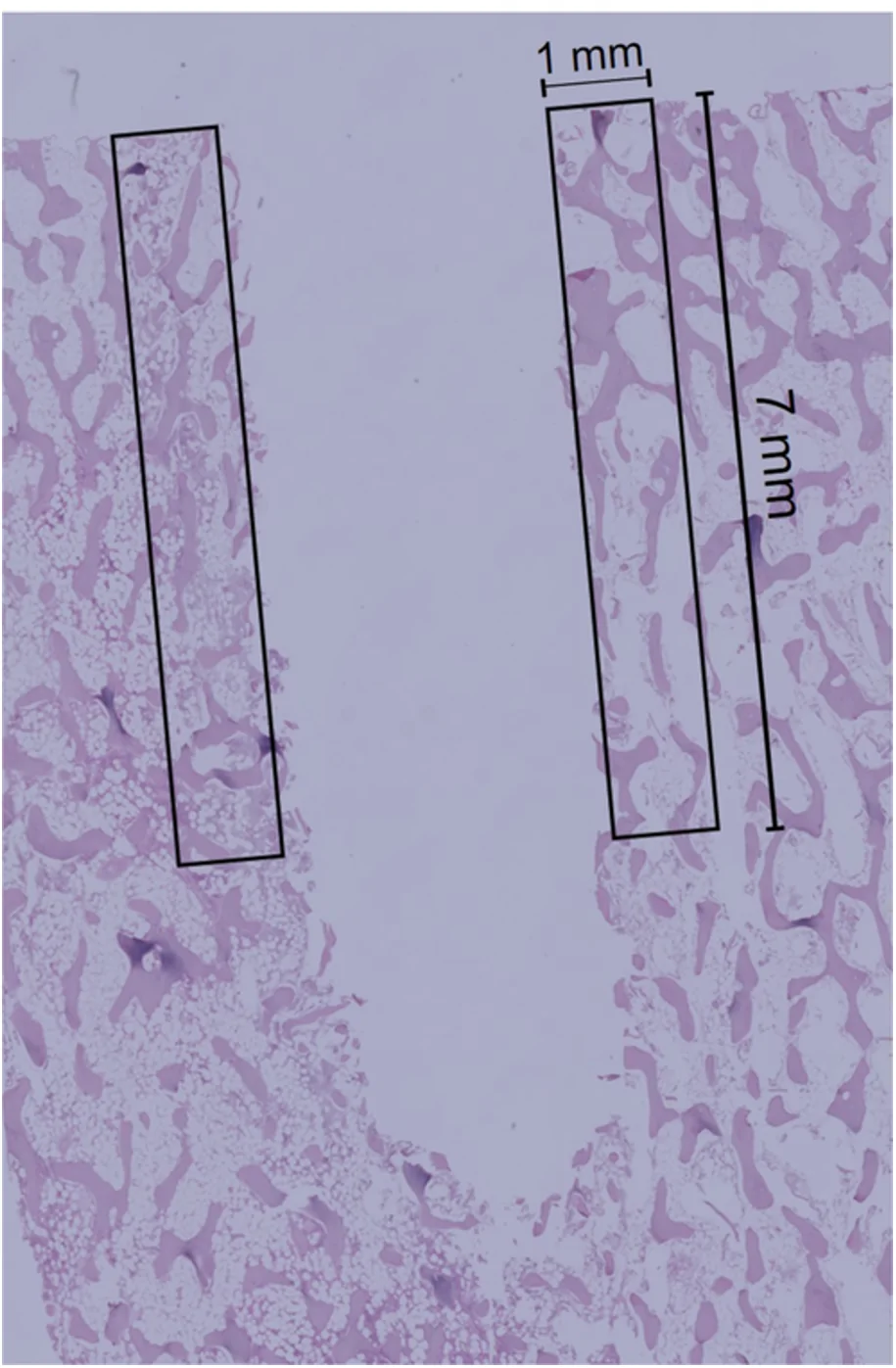
Region of interest (ROI).

After implant removal, decalcified thin sections were prepared and stained with hematoxylin and eosin. Histological analyses were performed using an optical microscope (BZ‐9000; Keyence, Osaka, Japan). The specimens were histomorphometrically analyzed using the ImageJ software (Fiji distribution; National Institute of Health, Bethesda, Maryland, USA). The region of interest (ROI) was defined as bone within 1 mm of the straight portion of the implant (Figure 3). The bone area fraction (BAF) was calculated as the ratio of bone area to total area.

ROI was defined as a 1 mm × 7 mm area of peri‐implant bone surrounding the straight portion of the implant.

### 2.6 Statistical Analysis

Statistical analysis was performed using unpaired *t*‐tests, Spearman's rank correlation coefficient, and linear regression analysis using Prism v7 (GraphPad, La Jolla, CA, USA). Statistical significance was set at *p* < 0.05.

## 3 Results

DTV ranged from 1.0 to 2.9 Ncm (mean 1.6 Ncm) and did not differ between the groups (undersized: 1.6 ± 0.5 Ncm, normal‐sized: 1.7 ± 0.5 Ncm). ITV was 12.2 ± 7.1 Ncm in the undersized group and 6.4 ± 3.7 Ncm in the normal‐sized group, showing a significant difference between groups. ISQ values were 56.4 ± 5.9 in the undersized group, and 54.1 ± 10.1 in the normal‐sized group, with no significant difference (Table 1).

**Table 1:** Measurement parameters.

	Undersized group	Normal‐sized group
DTV (Ncm)	1.6 ± 0.5	1.7 ± 0.5
ITV (Ncm)	12.2 ± 7.1	6.4 ± 3.7*
ISQ	56.4 ± 5.9	54.1 ± 10.1

*Note:* Mean values ± standard deviation (SD).

* *p* < 0.05

### 3.1 Correlations

**Figure 4:**
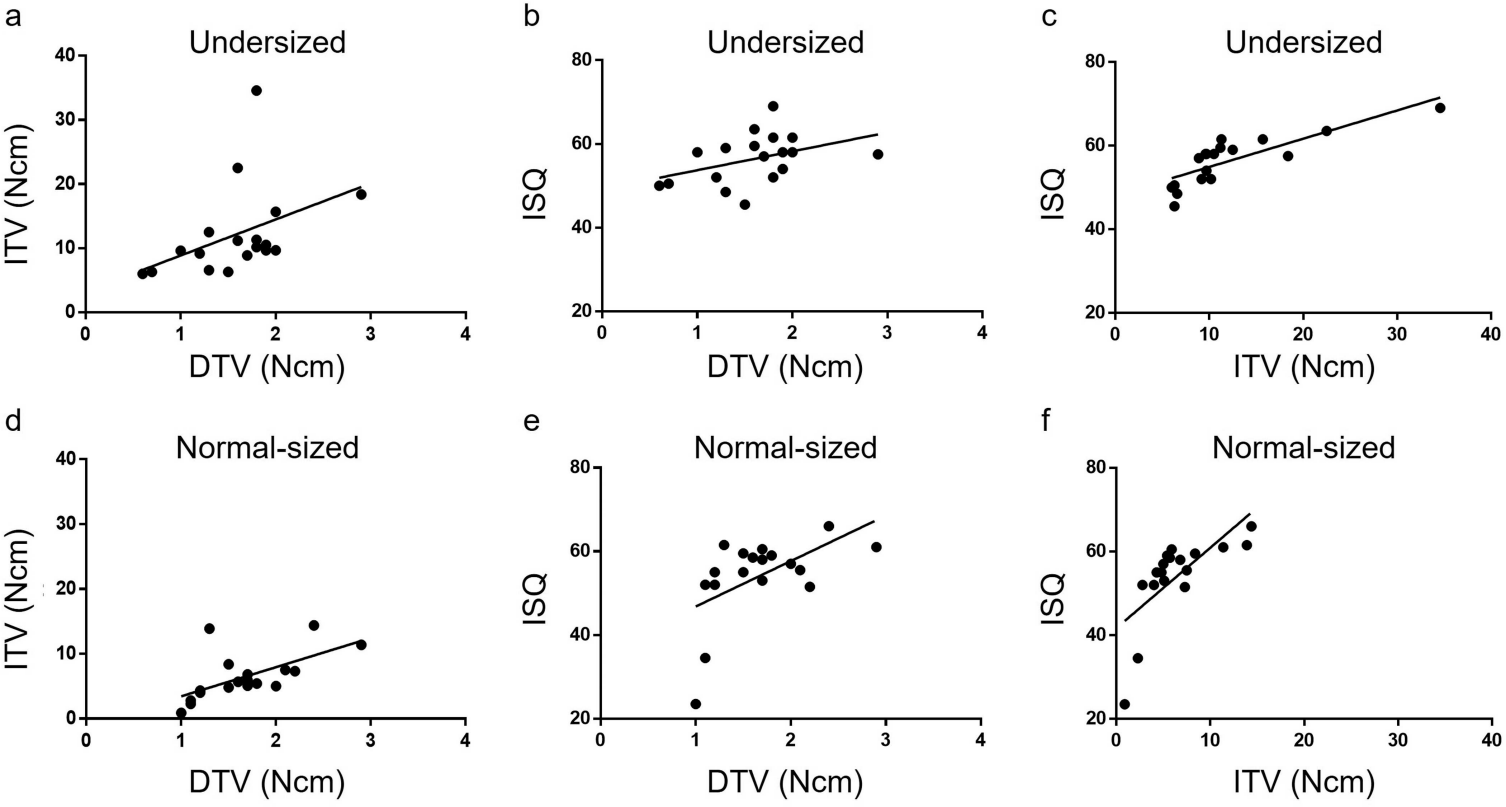
Correlations among DTV, ITV, and ISQ. (a) Correlation between DTV‐ITV in the undersized group (*r* = 0.6017). (b) Correlation between DTV‐ISQ in the undersized group (*r* = 0.4215, *p* = 0.0815). (c) Correlation between ITV‐ISQ in the undersized group (*r* = 0.8684). (d) Correlation between DTV‐ITV in the normal‐sized group (*r* = 0.7188). (e) Correlation between DTV‐ISQ in the normal‐sized group (*r* = 0.5308). (f) Correlation between ITV‐ISQ in the normal‐sized group (*r* = 0.8684).

A strong positive correlation was observed between the ITV and ISQ in both groups (undersized: *r* = 0.8684; normal‐sized: *r* = 0.8684) (Figure 4c,f). The DTV was positively correlated with the ITV in both the undersized (*r* = 0.6017) and normal‐sized (*r* = 0.7188) groups (Figure 4a,d). The DTV was also positively correlated with the ISQ in the normal‐sized group (*r* = 0.5308), but no significant correlation was found in the undersized group (*r* = 0.4215, *p* = 0.0815) (Figure 4b,e).

### 3.2 Histological Evaluation

**Figure 5:**
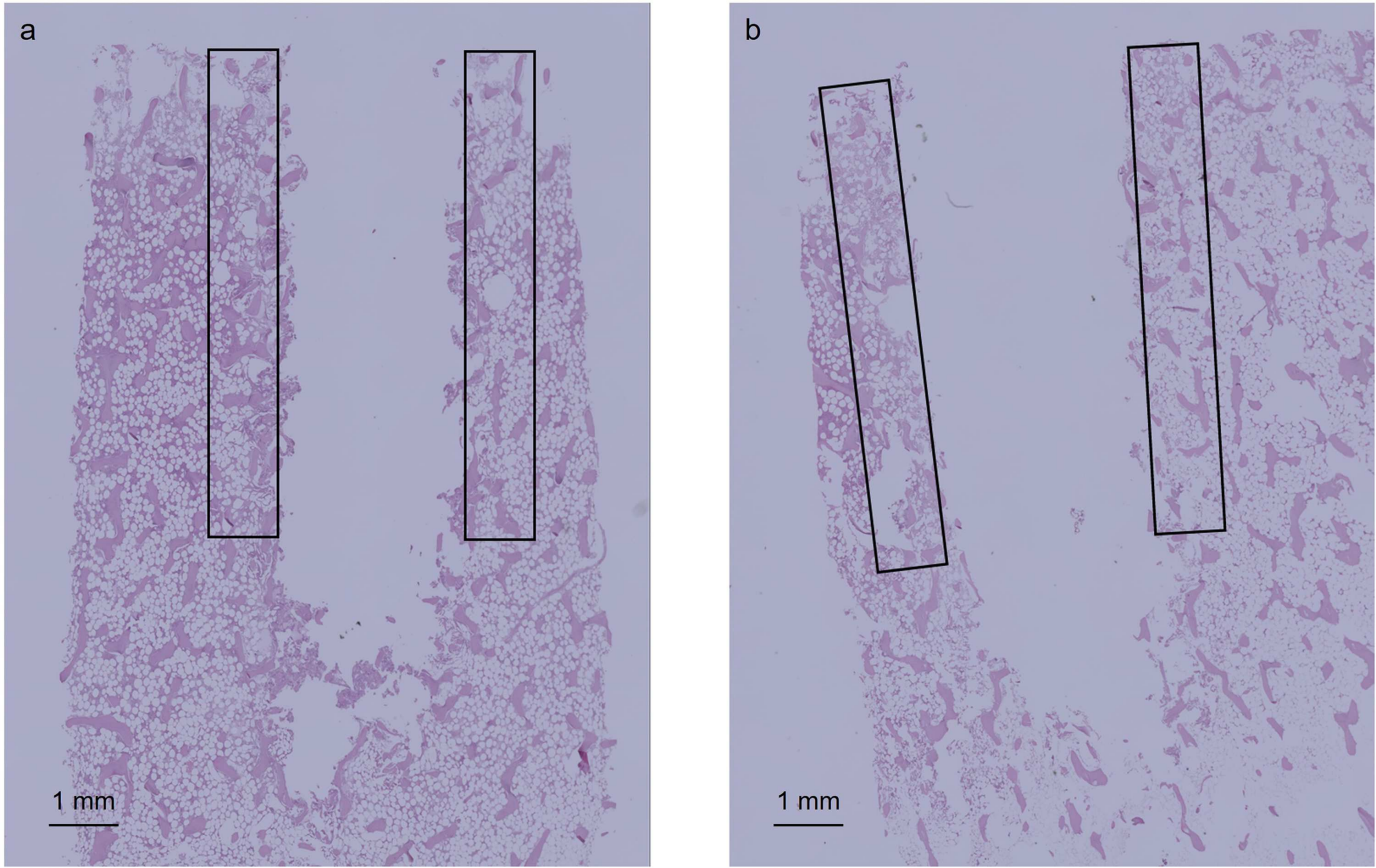
Histology. (a) Undersized group. (b) Normal‐sized group.

Histological observation revealed that the central implant socket was preserved in all specimens. The surrounding cancellous bone was well visualized, and no obvious differences in morphology were observed between the undersized and normal‐sized groups (Figure 5).

Representative hematoxylin and eosin‐stained sections are shown (scale bar, 1 mm). No significant differences were observed in the peri‐implant bone tissue between the two groups.

**Figure 6:**
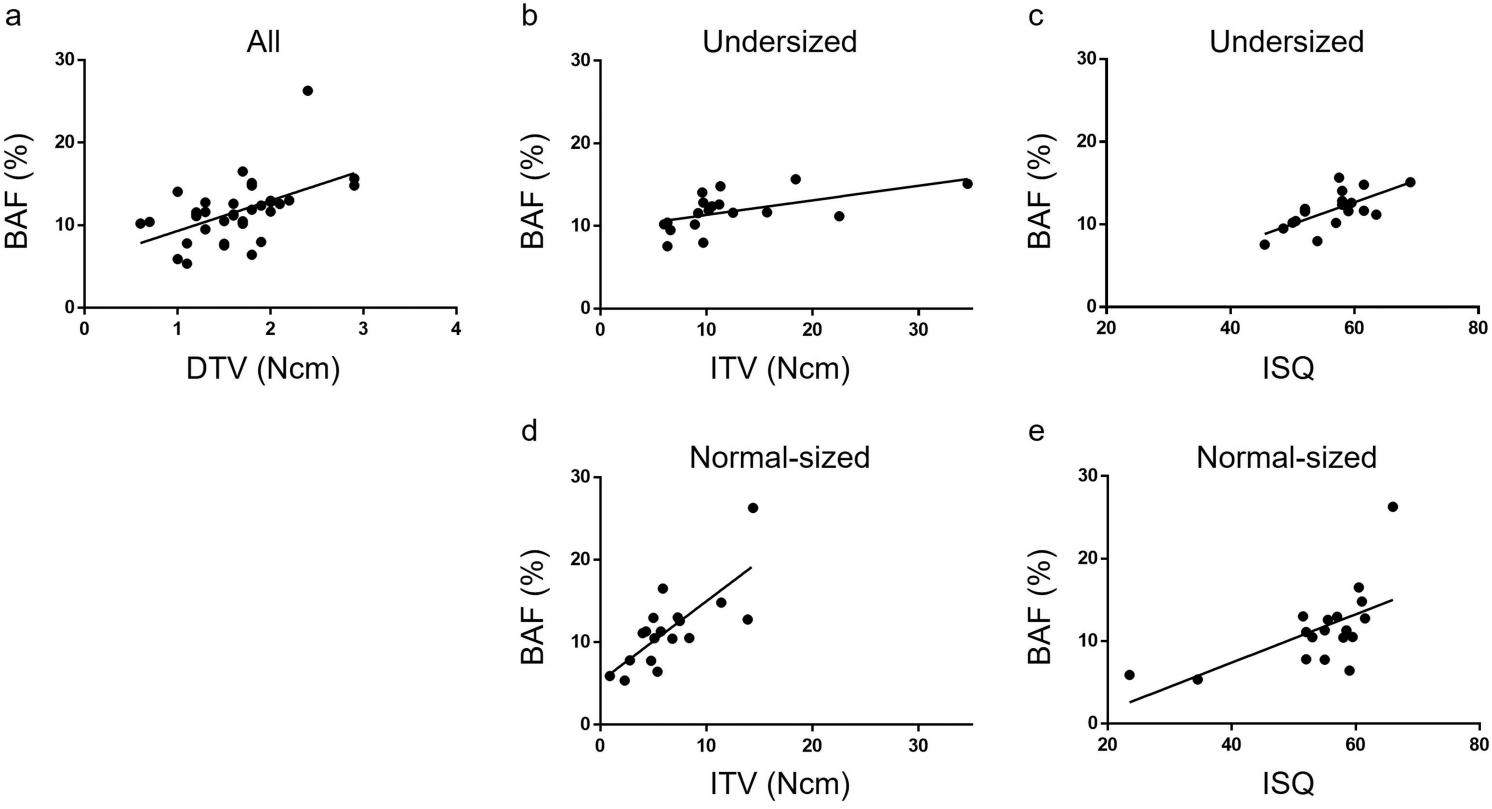
Correlation with BAF. (a) Correlation between DTV‐BAF in both groups (*r* = 0.5800). (b) Correlation between ITV‐BAF in the undersized group (*r* = 0.6488). (c) Correlation between ISQ‐BAF in the undersized group (*r* = 0.6460). (d) Correlation between ITV‐BAF in the normal‐sized group (*r* = 0.6987). (e) Correlation between ISQ‐BAF in the normal‐sized group (*r* = 0.5940).

BAF did not differ significantly between the groups, with values of 11.73 ± 2.27% in the undersized group and 11.52 ± 4.77% in the normal‐sized group. The DTV was positively correlated with BAF (*r* = 0.58) (Figure 6a). The ITV also demonstrated positive correlations with BAF in both groups (undersized: *r* = 0.6488; normal‐sized: *r* = 0.6987) (Figure 6b,d). Similarly, ISQ values positively correlated with BAF in both groups (undersized: *r* = 0.6460; normal‐sized: *r* = 0.5940) (Figure 6c,e).

## 4 Discussion

This study examined the correlation between DTV and primary stability at low bone density sites in biological cancellous bone and found that the undersized drilling protocol improved ITV and resulted in favorable primary stability. In cases with thick cortical bone, favorable primary stability is generally achieved owing to cortical engagement, and cortical thickness can be evaluated preoperatively using CT imaging (Al‐Juboori et al. 2024; Miyamoto et al. 2005). However, in cases with thin cortical bone, assessing the bone density of the cancellous region and obtaining adequate primary stability by selecting an appropriate drilling protocol are desirable (Marquezan et al. 2014).

In our previous study, we reported a strong correlation between DTV and CT values obtained using a bone density measurement drill.

DTVs have been reported to correlate with trabecular bone area and bone density. In the undersized drilling technique, implant manufacturers recommend drilling with a 2.8 mm diameter drill to place a regular‐sized implant (Alghamdi et al. 2011). Although torque detection generally becomes easier with increasing drill diameter, the present drill, despite its small diameter of 2.7 mm, allowed for the measurement of bone density prior to implant placement, even in the D4–D5 bone.

Bovine cancellous rib bone was used as the experimental material. Because the sternal side consists of costal cartilage, the vertebral side was selected. Additionally, the specimens were unfixed with formalin, allowing for the evaluation of both the DTV and ITV.

In the present study, bone density classification was performed using DTV in the cancellous portion of the bovine ribs, and low‐density regions (D4–D5) were selected.

A higher ITV was obtained in the undersized group than in the normal‐sized group. In both groups, DTV was positively correlated with ITV. These results are consistent with those of previous studies using artificial bone blocks (Wakamatsu et al. 2024). These results indicate that the drilling torque reflects the mechanical resistance of the bone and influences primary stability. Therefore, the DTVs measured intraoperatively are reflected in the ITVs, even in biological bone. This suggests that primary stability can be assessed by measuring DTV and that undersized preparations may improve primary stability in cases with low DTV.

In the present study, undersized preparations did not result in detectable differences in ISQ values compared to normal‐sized preparations. A positive correlation between DTV and ISQ was observed only in the normal‐sized group. Although a positive trend was observed in the undersized group, no significant correlation was found. In contrast, the ITV was positively correlated with the ISQ in both groups.

The significantly higher ITV observed in the undersized group than the normal‐sized group is attributable to greater compression of surrounding cancellous bone resulting in increased mechanical resistance during implant insertion. In contrast, no significant difference in ISQ was observed between the undersized and normal‐sized groups. This discrepancy may be explained by the different biomechanical principles underlying ITV and ISQ measurements. ITV primarily reflects the mechanical resistance encountered during implant insertion, whereas ISQ, obtained by resonance frequency analysis (RFA), reflects the stiffness of the implant–bone complex rather than the insertion resistance itself. As described by Sennerby and Meredith (2008), RFA applies a bending load to the implant that mimics functional loading and provides information on the stiffness of the implant–bone junction rather than the compressive forces generated during implant placement. Although ISQ has been reported to be affected by bone compression and the bone contact area (Yoon et al. 2011; Rosas‐Díaz et al. 2024; Huang et al. 2013; Park et al. 2011), previous studies have also demonstrated that implant‐bone contact is not necessarily correlated with RFA measurements (Akça et al. 2006; Ito et al. 2008).

The bovine bone used in this study was primarily D5 low‐density bone with sparse trabeculae. Therefore, although undersized preparations cause more bone compression forces during implant insertion, these localized mechanical changes were unlikely to substantially increase the overall stiffness of the implant‐bone complex. This is supported by the fact that there was no difference in the BAF between the two groups. Consequently, similar ISQ values were obtained despite the significantly higher ITV in the undersized group.

For histological evaluation, the ROI was set in the straight portion of the implant body because this region is considered to contribute the most strongly to primary stability, whereas the tapered portion mainly functions in bone cutting and guidance (Yamaguchi et al. 2015). Additionally, the bone within 1 mm of the implant primarily influences primary stability (Doi et al. 2021). This analysis evaluated the bone tissue immediately after implant placement without a healing period, and the measured BAF was therefore considered to reflect existing bone. In the present study, no difference in BAF was observed between the undersized and normal‐sized preparation groups, consistent with previous reports indicating no difference in bone volume attributable to drilling protocol differences in immediate post‐placement histological evaluations (Folkman et al. 2020; Giro et al. 2013). Although bone compression induced by drilling and implant placement may alter trabecular spacing, such changes are not necessarily detected as increased trabecular bone volume immediately after placement (Trisi et al.^2016^; Nagaraja et al. 2005). Considering that the trabecular bone structure in the present study was relatively sparse, the absence of a difference in BAF between the groups was considered reasonable.

The lack of difference in BAF immediately after placement indicates that the experimental conditions allowed for evaluation without inducing excessive bone destruction due to undersized preparation. Therefore, appropriate selection of drilling protocols based on DTV may allow for favorable primary stability while minimizing immediate damage to the bone structure. The BAF showed positive correlations with both ITV and ISQ in both groups, which is consistent with previous reports demonstrating the contribution of bone volume to primary stability (Marquezan et al. 2012; Merheb et al. 2018). Although no healing period was included in this study, and the evaluated BAF reflected existing bone, the observed correlations suggest that immediate mechanical primary stability is influenced by existing bone volume. Furthermore, the BAF was positively correlated with the DTV. Although the region in which the DTV was measured was not included in the histological ROI, this result suggests that DTV measurement may serve as an indicator of existing bone volume in low‐density bone regions.

In clinical practice, cases with thin cortical bone that rely primarily on cancellous bone support may present difficulties in achieving primary implant stability when cancellous bone density is low. If the bone quality at the implant site can be directly and objectively assessed intraoperatively, appropriate implant socket preparation protocols can be selected before implant placement. In this study, a positive correlation was observed between the BAF and DTV, suggesting that intraoperative measurement of DTV may allow for the assessment of bone volume at low‐density implant sites. By directly and objectively evaluating bone density before implant socket preparation, it may be possible to select implants and drilling protocols that facilitate favorable primary stability. This approach may contribute to shortened healing periods and a reduced risk of implant failure. Therefore, this method may be useful as an intraoperative indicator of localized bone characteristics that are difficult to evaluate using preoperative imaging or tactile sensation.

This study employed an immediate placement‐removal model without a healing period; therefore, only the mechanical aspects of primary stability were evaluated, and the biological healing process and subsequent osseointegration could not be assessed. BAF reflects pre‐existing trabecular bone architecture but does not present the biological response to different drilling protocols. Therefore, the improved primary stability observed with the undersized drilling protocol should not be interpreted as evidence of superior long‐term clinical success. Although undersized preparation may enhance primary mechanical stability, excessive bone compression has been reported to induce cellular damage and potentially impair subsequent bone remodeling and osseointegration. These biological effects were beyond the scope of the present ex vivo study. Furthermore, this study was limited to a specific bone density (D4–D5), implant system, and absent of cortical bone. In clinical practice, cortical bone engagement is an important determinant of primary implant stability, and cortical bone morphology can be assessed preoperatively using computed tomography. The present experimental model was intentionally designed to isolate the influence of cancellous bone by eliminating the confounding effect of cortical bone. Therefore, the model does not fully reproduce physiological clinical conditions, and the measured implant stability values may differ from those observed in vivo.

Accordingly, caution should be exercised when extrapolating the present findings to clinical practice.

Although the proposed measurement drilling protocol represents an additional intraoperative step, the measurement requires only a few seconds during the initial stages of the drilling protocol and is not intended for routine use in every implant surgery. Instead, it may serve as an adjunctive method when an objective assessment of cancellous bone quality is clinically desirable. A major advantage of this approach is that it quantitatively evaluates drilling resistance, thereby complementing the surgeon's tactile perception with an objective numerical parameter. This may be particularly beneficial for less experienced clinicians and for educational purposes, where tactile sensation is inherently subjective and difficult to standardize. In the present study, the measurement was performed using an implant motor with a preset torque limit to minimize excessive torque loading during low‐speed drilling. If its clinical utility is confirmed, the small increase in procedural time may be justified by the additional objective information available before implant placement.

Future studies using models with healing periods and clinical investigations are required to determine whether DTV‐guided implant site preparation can improve treatment planning, optimize drilling protocols, and contribute to predictable long‐term implant outcomes.

## 5 Conclusion

This study revealed that DTV reflects the existing bone volume in low‐density tissue and correlates with primary implant stability. Intraoperative drilling torque assessment may facilitate the selection of appropriate drilling protocols to achieve primary stability while minimizing bone damage, providing a clinically useful method for evaluating low‐density implant sites.

## Author Contributions

Kazuya Doi contributed to the study concept and design. Yusuke Morimoto participated in the data collection, and Reiko Kobatake and Kazuya Doi checked the data. Reiko Kobatake performed the statistical analyses. Yusuke Morimoto, Reiko Kobatake, and Kazuya Doi drafted the manuscript. Yusuke Morimoto, Reiko Kobatake, Kazuya Doi, Yoshifumi Oki, Kaien Wakamatsu, Tomoko Izumikawa, and Kazuhiro Tsuga reviewed the findings of data analyses, participated in writing the manuscript and approved the final draft of the manuscript submitted to the journal.

## Conflicts of Interest

The authors declare no conflicts of interest.

## Data Availability

The data that support the findings of this study are available from the corresponding author upon reasonable request.
